# Structures of KaiC Circadian Clock Mutant Proteins: A New Phosphorylation Site at T426 and Mechanisms of Kinase, ATPase and Phosphatase

**DOI:** 10.1371/journal.pone.0007529

**Published:** 2009-11-26

**Authors:** Rekha Pattanayek, Tetsuya Mori, Yao Xu, Sabuj Pattanayek, Carl H. Johnson, Martin Egli

**Affiliations:** 1 Department of Biochemistry, Vanderbilt University, School of Medicine, Nashville, Tennessee, United States of America; 2 Department of Biological Sciences, Vanderbilt University, Nashville, Tennessee, United States of America; Institute of Molecular and Cell Biology, Singapore

## Abstract

**Background:**

The circadian clock of the cyanobacterium *Synechococcus elongatus* can be reconstituted *in vitro* by three proteins, KaiA, KaiB and KaiC. Homo-hexameric KaiC displays kinase, phosphatase and ATPase activities; KaiA enhances KaiC phosphorylation and KaiB antagonizes KaiA. Phosphorylation and dephosphorylation of the two known sites in the C-terminal half of KaiC subunits, T432 and S431, follow a strict order (TS→pTS→pTpS→TpS→TS) over the daily cycle, the origin of which is not understood. To address this void and to analyze the roles of KaiC active site residues, in particular T426, we determined structures of single and double P-site mutants of *S. elongatus* KaiC.

**Methodology and Principal Findings:**

The conformations of the loop region harboring P-site residues T432 and S431 in the crystal structures of six KaiC mutant proteins exhibit subtle differences that result in various distances between Thr (or Ala/Asn/Glu) and Ser (or Ala/Asp) residues and the ATP γ-phosphate. T432 is phosphorylated first because it lies consistently closer to Pγ. The structures of the S431A and T432E/S431A mutants reveal phosphorylation at T426. The environments of the latter residue in the structures and functional data for T426 mutants *in vitro* and *in vivo* imply a role in dephosphorylation.

**Conclusions and Significance:**

We provide evidence for a third phosphorylation site in KaiC at T426. T426 and S431 are closely spaced and a KaiC subunit cannot carry phosphates at both sites simultaneously. Fewer subunits are phosphorylated at T426 in the two KaiC mutants compared to phosphorylated T432 and/or S431 residues in the structures of wt and other mutant KaiCs, suggesting that T426 phosphorylation may be labile. The structures combined with functional data for a host of KaiC mutant proteins help rationalize why S431 trails T432 in the loss of its phosphate and shed light on the mechanisms of the KaiC kinase, ATPase and phosphatase activities.

## Introduction

In the cyanobacterium *Synechococcus elongatus* the KaiC, KaiA and KaiB proteins form a minimal circadian clock *in vivo* that is able to sustain a ca. 24-hour period in the absence of a transcription-translation oscillatory feedback loop [1]. Remarkably, the clock can be reconstituted *in vitro* with just the three Kai proteins and ATP [2]. The *in vitro* timer displays the hallmarks of all circadian oscillators, namely a period of approximately 24 hours, tuned to the daily light-dark cycle, and temperature compensation [3]. The discovery of this *in vitro* oscillator paves the road to a rigorous biochemical, biophysical and structural characterization of a molecular clock [4].

KaiC comprises the core of the clock and acts as a kinase, phosphatase and ATPase [5]–[8]. KaiA enhances KaiC phosphorylation and in its absence *in vitro*, KaiC dephosphorylates over time, and KaiB antagonizes KaiA action [6], [7], [9]–[11]. Three-dimensional structures of the full-length cyanobacterial KaiA, KaiB and KaiC proteins have been reported during the past five years (reviewed in refs. [12] and [13]). KaiC is the result of a gene duplication [14] and forms a homo-hexamer of ca. 360 kDa molecular weight [15], [16]. The *kaiC* gene displays similarities to the *recA* and *dnaB* families [14], but a helicase activity for KaiC has not been established despite intense efforts [17]. The crystal structure of KaiC from *S. elongatus* revealed a hexamer in the shape of a double doughnut with approximate dimensions 100×100 Å, whereby the N-terminal CI and C-terminal CII halves of subunits are joined by a 15-amino acid linker [18]. A total of twelve ATP molecules are bound between subunits in the upper and lower rings and C-terminal peptide tails that protrude from the dome-shaped surface of the CII hexamer give the KaiC double-doughnut an asymmetric appearance [19]. Both the KaiA and KaiB dimers contact only the KaiCII half and hybrid structural techniques have recently yielded 3-dimensional models of the KaiAC [19] and KaiBC complexes [20] and provided insights into the modes of action of the KaiA and KaiB proteins.

Two phosphorylation sites (P-sites), T432 and S431 that are both located in the CII half were identified in KaiC [21], [22]. Over a 24-hour cycle phosphorylation proceeds in a strict order TS→pTS→pTpS→TpS→TS [23], [24]. Rapid and repeated association of KaiA with KaiC results in the conversion from the hypo-phosphorylated (TS) to the hyper-phosphorylated (pTpS) form [25], [26]. KaiB binds preferably to the hyper-phosphorylated form and reverses KaiA's action, whereby first T432 and then S431 are being dephosphorylated [23]–[26]. KaiB binding and dephosphorylation are accompanied by the exchange of KaiC subunits [25], a mechanism that is crucial to maintaining a stable oscillator [26], [27]. Rather than individual KaiC particles engaging in various protein-protein associations and moving essentially in lockstep from the hypo- to the hyper- and back to the hypo-phosphorylated form, the cyanobacterial minimal timer is characterized by a mixture of oscillating populations of free KaiC, KaiA and KaiB proteins and KaiAC and KaiBC as well as KaiABC complexes of different concentrations [25], [26].

**Figure 1:**
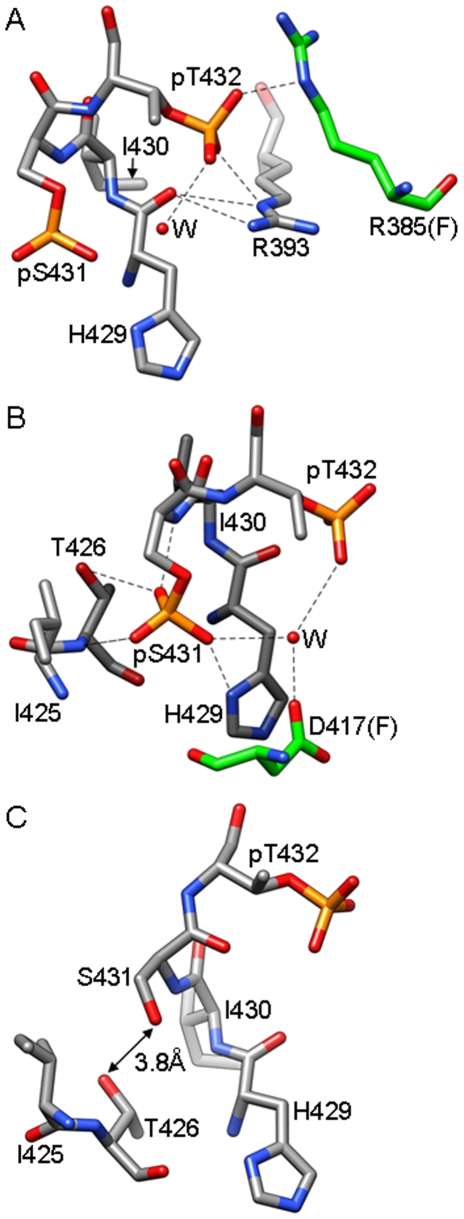
Inter- and intra-subunit interactions of phosphorylated Thr and Ser residues in KaiC. (**A**) Formation of a salt bridge between pT432 (A subunit) and R385 (F subunit; carbon atoms highlighted in green). Hydrogen bonds are dashed lines. (**B**) Interactions of pS431 (A subunit) with T426, H429 and D417 (F subunit, carbon atoms highlighted in green). (**C**) Configuration of T426 and S431 in the C subunit that lacks phosphorylation at S431. The side chain hydroxyls are too far removed to engage in a hydrogen bond.

Recent reports on KaiC and the mechanism of the KaiABC circadian clock take into account only two P-sites. However, we also found the T426A mutant ( = a426/S431/T432 = Kai^aST^ = aST) to be arhythmic [21]. In the crystal structure S431 and T426 are very tightly spaced and the side chain of T426 engages in a H-bond interaction with S431 when the latter is phosphorylated [21] (**Fig. 1**). We established that mutations of T426 alter the KaiC phosphorylation profiles *in vivo* and that residue 426 needs to be phosphorylatable and not simply capable of forming a H-bond to pS431 [28]. Moreover, like the KaiC^aST^ mutant, nST as well as eST abolish rhythmicity in strains expressing these mutants alone. Interestingly, when T426-mutant KaiCs are co-expressed with the wt enzyme, aST exhibits a dominant negative effect, whereas strains co-expressing either nST or eST with wt-KaiC show significantly longer periods of around 30 hours.

To analyze the phosphorylation patterns of KaiC P-site mutants and to visualize potential conformational variations in the vicinity of bound ATP and residues 432 and 431 at hexamer subunit interfaces, we determined crystal structures of the *S. elongatus* KaiC single mutants T432A (TSa), S431A (TaT), S431D (TdT), and T426N (nST) and the double mutants T426A/T432A (aSa) and S431A/T432E (Tae). The combined structural data expose subtle changes in the orientations of the H423-I433 loop region harboring phosphorylated residues relative to the γ-phosphate of ATP compared with the structure of wt-KaiC. The structures of the Tae and TaT mutants reveal that T426 residues in some of the six subunits carry a phosphate group and call into question the common assumption of just two P-sites in the core clock protein. Beyond the discovery of a third P-site in KaiC, the structures of mutants also provide insight into the mechanisms of the kinase, phosphatase and ATPase activities and the role of individual residues, including T426 in the catalytic processes.

## Results

### Crystal Structures of *S. elongatus* KaiC Single- and Double-Mutant Proteins

**Figure 2:**
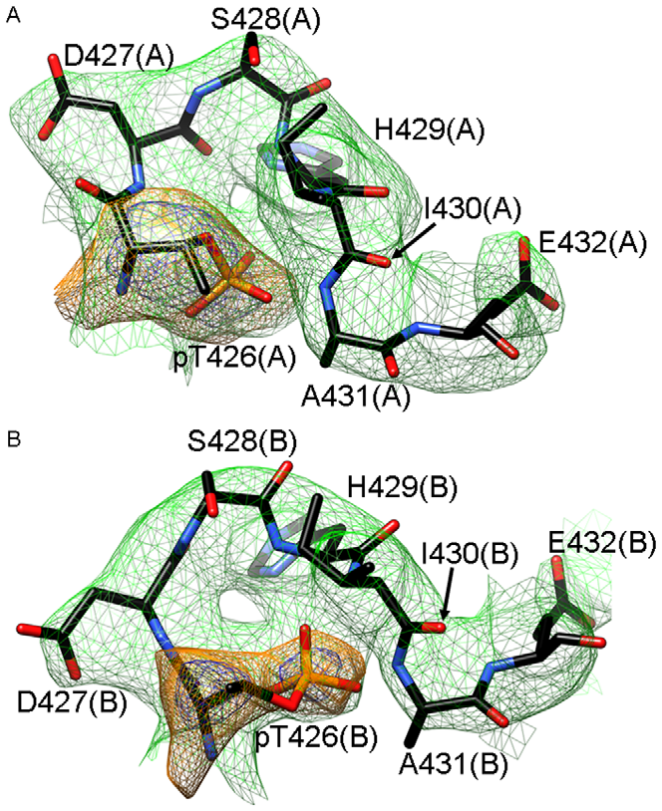
Example of the quality of the electron density. Final Fourier sum (2F*o*–F*c*) electron density (1σ, green) in the region of the P-site loop (residues pT426 to E432) in A, the subunit A, and B, in the B subunit, in the structure of KaiC^Tae^. Annealed omit (2F*o*–F*c*) electron density around residue T426 is depicted in magenta (4.5σ threshold) and orange (3σ threshold).

We determined crystal structures of the full-lengths TSa, TaT, TdT, nST, aSa and Tae KaiC mutant proteins at resolutions of between 2.9 and 3.3 Å (**Table 1**). All mutant proteins were expressed with a C-terminal His_6_ tail that was not removed for crystallization. The structures are homologous to that of wt-KaiC and diffraction data were phased using the molecular replacement technique and the KaiC structure with PDB ID code 3DVL [4], [19]. Following rigid-body, simulated annealing and individual atom and B-factor refinement cycles, Fourier sum (2F_o_–F_c_) and difference (F_o_–F_c_) electron density maps were computed and inspected for >3σ peaks around Ser and Thr side chains. Even at the resolutions of the structures reported here, visualization of phosphorylation sites is typically straightforward. Examples of the omit electron density around T426 in the structure of the Tae double mutant following partial refinement and the quality of the final electron density are depicted in **Fig. 2**.

**Table 1:** Selected crystal data^a^ and refinement parameters for structures of *S. elongatus* KaiC mutant proteins^b^.

Mutant structure	TSa	TaT	TdT	nST	aSa	Tae
Space group	*P*2_1_2_1_2_1_	*P*2_1_2_1_2_1_	*P*2_1_2_1_2_1_	*P*2_1_2_1_2_1_	*P*2_1_2_1_2_1_	*P*2_1_2_1_2_1_
Unit cell *a* [Å]	132.30	133.23	132.50	133.66	132.93	132.28
*b* [Å]	135.11	134.96	135.83	135.51	135.42	135.03
*c* [Å]	204.50	204.88	204.32	204.53	204.62	204.47
Resolution [Å]	2.9	3.2	3.2	3.2	3.0	3.3
Completeness [%]	93.8	93.4	91.5	92.9	99.9	98.3
Outer shell [%]	84.2	57.2	86.6	81.2	99.9	88.7
Resol. range [Å]	3.0–2.9	3.3–3.2	3.3–3.2	3.3–3.2	3.1–3.0	3.4–3.3
I/σ(I) (outer shell)	17.8(2.8)	10.7(1.0)	16.8(4.4)	14.7(2.8)	23.4(2.7)	13.8(2.3)
R-merge [%]	7.2	8.5	9.0	7.0	6.1	10.3
Outer shell [%]	56.4	55.5	52.0	48.7	42.7	57.4
R-work [%]	22.8	24.2	23.3	23.7	22.9	23.1
R-free [%]	28.2	30.7	29.6	31.0	28.8	26.9
Reflections used for R-free [%]	8.4	5.4	8.3	7.2	8.9	8.3
Number of ATP molecules	12	12	12	12	12	12
No. of atoms	23,356	23,919	23,856	23,899	23,930	23,916
R.m.s.d bonds [Å]	0.009	0.01	0.009	0.009	0.008	0.009
R.m.s.d angles [°]	1.4	1.5	1.5	1.5	1.5	1.4

a All data were collected on either the 21-ID-F or 21-ID-G beamlines at the Advanced Photon Source (Argonne National Laboratory, Argonne, IL), using MAR225 or MAR300 CCDs. The data collection temperature was 110K.

b KaiC mutations xyz or mutants KaiC^xyz^ are sequentially shown in order of sites x = 426, y = 431, and z = 432 where the wild-type residue is in upper case and mutated residues are shown in lower case, i.e. Tsa or KaiC^TSa^, where the residue at position 426 is T, the residue at position 431 is S, and the residue at position 432 is mutated to A.

### Phosphorylation States of the Wild Type and Mutant KaiC Proteins

**Figure 3:**
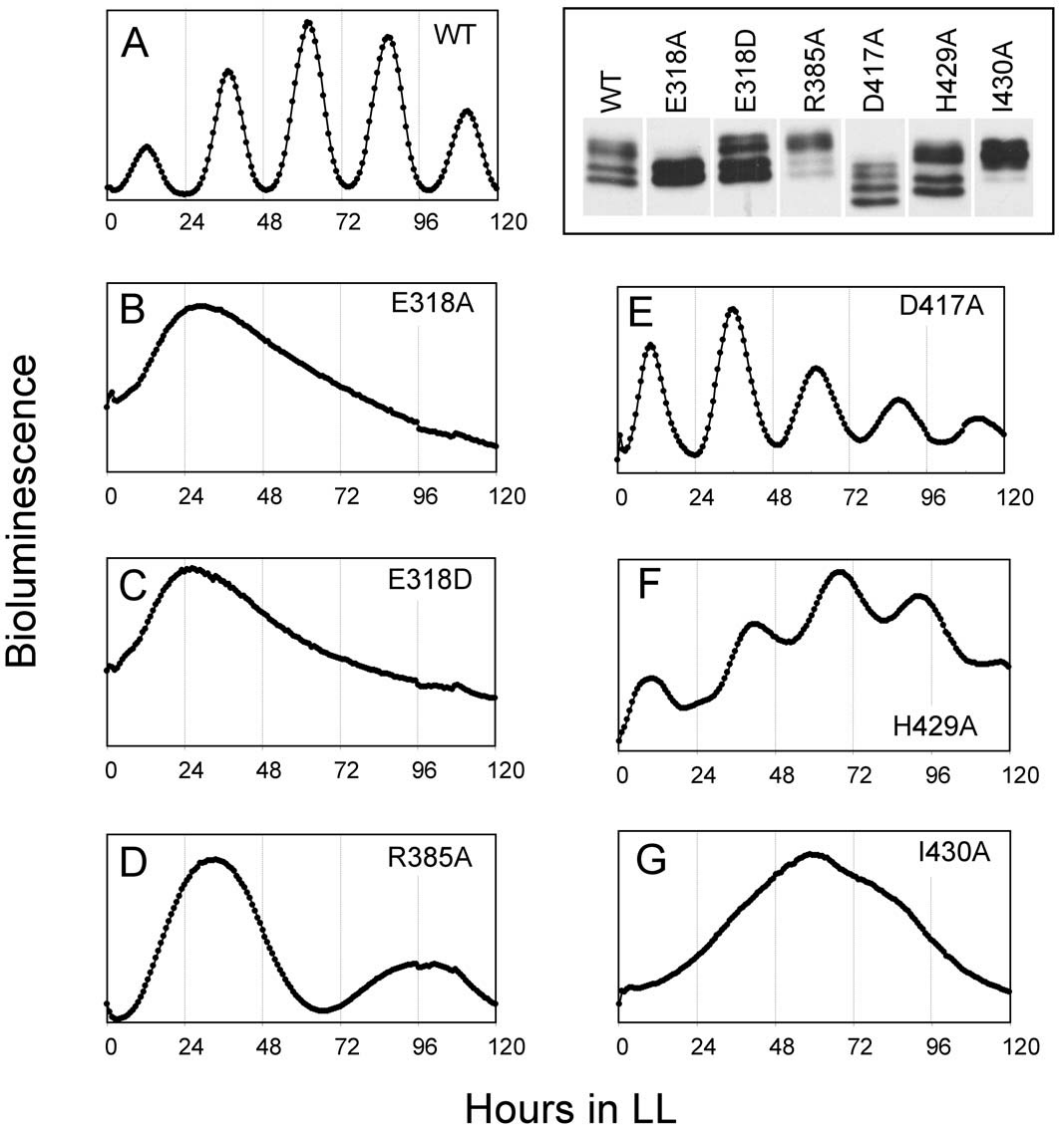
Mutations in the inter- and intra-subunit neighborhood of the P-sites affect *in vivo* rhythmicity and KaiC phosphorylation. Bioluminescence profiles of P*kaiBC*::*luxAB* reporter strains carrying (**A**) wild-type KaiC or amino acid substitutions at (**B**) E318A, (**C**) E318D, (**D**) R385A, (**E**) D417A, (**F**) H429A, or (**G**) I430A in KaiC. The mutations either at E318A, E318D or I430A abolished the circadian rhythmicity, whereas the mutants R385A, D417A, or H429A exhibited promoter activity rhythms. The circadian periods of these bioluminescence rhythms were 36∼48 h (R385A), 25.6 h (D417A), 28.0 h (H429A) and 24.8 h (WT; Wild-Type), respectively. The cells expressing the wild-type or mutant KaiCs were harvested at ZT4 and extracts were analyzed by immunoblot with anti-KaiC antibodies (right top). Therefore, these phosphorylation patterns are the *in vivo* patterns at ZT4.

An important consequence of the phosphorylation at T432 and S431 in the KaiCII half is the formation of additional interactions between residues at the subunit interface [21] that we have suggested to have the effect of unidirectional phosphorylation reactions driving the oscillator forward [4]. In the structure of wt-KaiC, the T432 residues in all six subunits are phosphorylated and form a salt bridge with R385 from adjacent subunits (**Fig. 1A**, **Table 2**). In the four subunits that exhibit phosphorylation at S431, the phosphates are within hydrogen bonding distance from H429 from the same subunits, and His itself engages in a stacking interaction with D417 from the adjacent subunit (**Fig. 1B**). Phosphorylation is therefore expected to stabilize the subunit interface relative to the hypo-phosphorylated form, whereby the pT432…R385 interaction supposedly makes the chief contribution because it stitches together two charged residues across the interface. This view is supported by the long-period phenotype of the R385A mutant (>40 hours, **Fig. 3**); KaiC^TSa^ itself is arhythmic [21]. By comparison, the pS431…H429 interaction is intra-subunit but may influence the inter-subunit H429…D417 interaction, i.e. via a change in the protonation state of histidine. However, the period of the H429A mutant is increased only modestly (28 hours) and the D417A mutant displayed a normal period (**Fig. 3**); the KaiC^TaT^ mutant is arhythmic as established earlier [21]. More importantly, phosphorylation of S431 leads to a new interaction with T426 (**Fig. 1B**), whereas in the unphosphorylated state (subunits C and D, **Fig. 1C**), S431 and T426 are spaced somewhat too far apart to allow formation of a hydrogen bond. Interestingly, like the TSa and TaT mutants KaiC^aST^ is arhythmic [21].

**Table 2:** Phosphorylation patterns in crystal structures of *S. elongatus* KaiC mutant proteins and distances between phosphorylation sites and ATP.

KaiC protein	No. of phosphates in CII half			Avg. distance in Å to Pγ (ATP)^a^		
P-site	432	431	426	432	431	426
Wild Type	6	4	–	8.2 (5.6)	9.4 (7.8)	12.5 (13.5)
TSa	–	6	–	5.6 (5.3)	9.1 (7.8)	11.9 (13.5)
TaT	6	–	1	8.2 (5.5)	7.6 (7.7)	12.3 (13.6)
TdT	3	–	–	8.3 (5.8)	9.1 (8.2)	12.5 (13.7)
nST	6	3	–	7.9 (5.4)	8.8 (7.7)	11.9 (13.9)
aSa	–	6	–	5.5 (5.5)	7.9 (7.8)	12.8 (13.5)
Tae	–	–	4	7.6 (5.6)	7.4 (7.6)	11.9 (13.4)

a Distances between Pγ (ATP) and P (pThr, pSer)/Oγ (Thr)/Cβ (Ala)/Cγ (Asp, Asn)/Cδ (Glu); distances between Cα and Pγ are listed in parentheses.

The number and distribution of phosphorylation sites in the individual KaiC mutants are summarized in **Table 2**. This table also lists the distances in the wt-KaiC and mutant structures between the γ-phosphorus of ATP and either the Cα positions or selected side chain atoms [i.e. P (pThr, pSer), Oγ (Thr), Cβ (Ala), Cγ (Asp, Asn), or Cδ (Glu)] of residues 432, 431 and 426. If not mentioned otherwise the distances given in the text refer to side chain atoms of these three residues. The TSa and the TaT mutants carry six phosphates on S431 and T432, respectively. Thus, although T432 is the primary phosphorylation site, its mutation to Ala does not prevent a phosphate from being transferred to S431. This observation supports the notion that in wt-KaiC, T432 and S431 get sequentially phosphorylated via the same mechanism. When T432 is not available (Ala mutant), the ATP γ-phosphate is directly transferred to S431. An alternative kinase mechanism that could readily explain the order of phosphorylation (T432 first and S431 second) would entail an initial transfer of the phosphate to T432 that then hands it off to S431 before being phosphorylated itself a second time. However, this is not the case in KaiC and T432 likely receives the phosphate first because it is closer to the γ-phosphate than S431 (8.2 Å vs. 9.4 Å on average in wt-KaiC). This conclusion is also in line with the observation that S431 carries a phosphate in the TSe mutant [23].

Interestingly, in the structure of KaiC^TSa^, A432 has moved closer to the γ-phosphate group of ATP: average distance 5.6 Å vs. 8.2 Å (wt-KaiC). One may have expected the above KaiC^TSa^ mutant to represent the state before dephosphorylation of the second P-site (pS431). Instead its structure is more representative of the initial phosphorylation event. By comparison, in the structure of KaiC^TaT^, the average distances between the γ-phosphate and the pT432 and A431 residues (side chains, **Table 2**) appear quite similar at first sight. This would not be unexpected: once T432 residues have become phosphorylated, S431 residues (A431 in the mutant) will shift into the active site to receive their phosphate. However, A431 residues in the structure of KaiC^TaT^ remain farther removed from the γ-phosphate of ATP (7.7 Å on average; Cα…Pγ) than A432 residues in the structure of KaiC^TSa^ (5.3 Å on average).

The structures of the TdT, nST and aSa mutants provide further support for the conclusion that T432 is the principal phosphorylation site because it lies closest to the γ-phosphate. In all three structures, residue 432 is distinctly closer to the γ-phosphate than 431, namely between 0.8 and 2.4 Å on average (**Table 2**). The difference in distance is smallest in the case of KaiC^TdT^; this mutant was studied as a model for the second step of dephosphorylation, with T432 residues having lost their phosphates and pS431 residues about to give up theirs. Indeed, of all structures in which T432 could potentially be phosphorylated, KaiC^TdT^ is the one that shows the lowest phosphorylation level of T432 (phosphorylated in three subunits). Nevertheless the phosphorylation levels and average distances to the ATP γ-phosphate of KaiCII 432 and 431 residues corroborate the idea that the former is the principal phosphorylation site mainly because of its proximity to ATP. In fact the spacing is particularly tight in the structures of the TSa and aSa mutants. There the average Cβ…Pγ distances (5.6 Å and 5.5 Å, respectively) exceed by less than 1 Å the sum of the van der Waals radii of the methyl and phosphate groups (4.8 Å).

**Figure 4:**
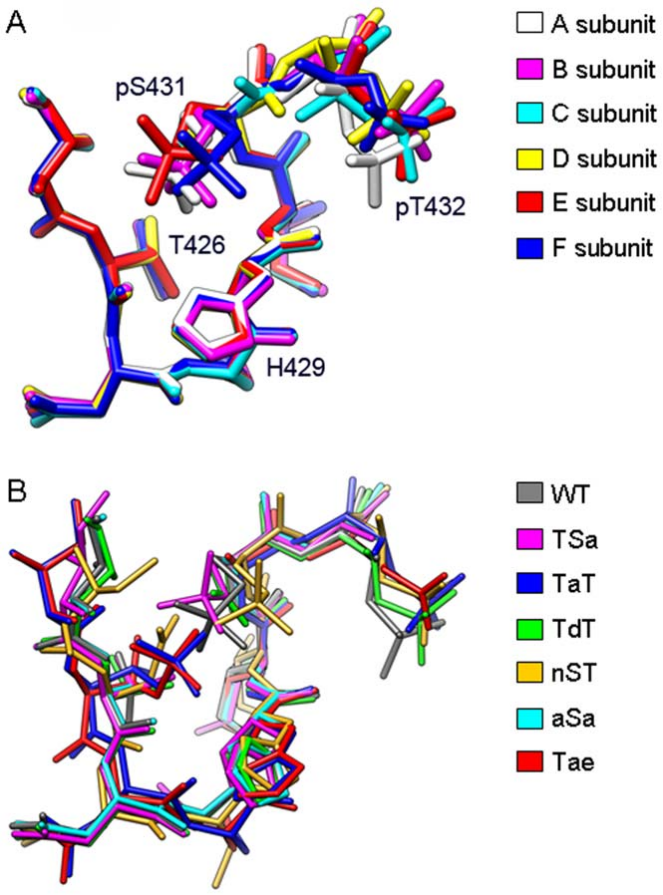
Conformational variations in the P-site loop region (residues 425 to 432) in wt- and mutant-KaiCs. (**A**) Superimposition of the P-site loops from all six subunits in the structure of wt-KaiC. A total of 505 atoms from subunit A were included to compare its geometry to those of the other five subunits, using the Chimera MatchMaker tool with the Smith-Waterman algorithm (BLOSUM-N matrix). With respect to the A subunit the B, C, D, E, and F subunits had r.m.s.d.'s of 0.25 Å, 0.29 Å, 0.30 Å, 0.31 Å, and 0.26 Å, respectively. (**B**) Superimposition of selected P-site loops in subunits A from structures of KaiC mutant proteins relative to wt-KaiC. The procedure was identical to that used for generating the superimposition in panel A and the r.m.s.d.'s are: TSa (484 atoms; 0.27 Å), TaT (506 atoms; 0.36 Å), TdT (506 atoms; 0.25 Å), nST (505 atoms; 0.51 Å), aSa (506 atoms; 0.14 Å) and Tae (506 atoms; 0.36 Å).

From the distance data in **Table 2** it is clear that different phosphorylation states do not correlate with drastic conformational changes in the P-site loop region. In particular, the conformations of the loop with both T432 and S431 phosphorylated and with just T432 carrying a phosphate are quite similar. This is illustrated by a superimposition of the loop regions from the six subunits in the structure of wt-KaiC (**Fig. 4A**). Although we do not have a structure of the non-phosphorylated form of KaiC at this time (or of the KaiC^Taa^, KaiC^aaa^, and KaiC^aST^ mutants), we do not expect the conformations of the hypo- and hyper-phosphorylated forms to be drastically different. In fact a superimposition of subunits from the six KaiC mutant structures reveals only minor adjustments in the geometry of the P-site loop region (i.e. in the spacing of the S431 and T426 residues, **Fig. 4B**). Thus, the combined structural data argue against a mechanism underlying the rhythmic transformation from the hypo- to the hyper- and back to the hypo-phosphorylated form that would require large conformational adjustments in the region harboring the phosphorylation sites.

### The Structures of the KaiC TaT and Tae Mutants Reveal a Third Phosphorylation Site

**Figure 5:**
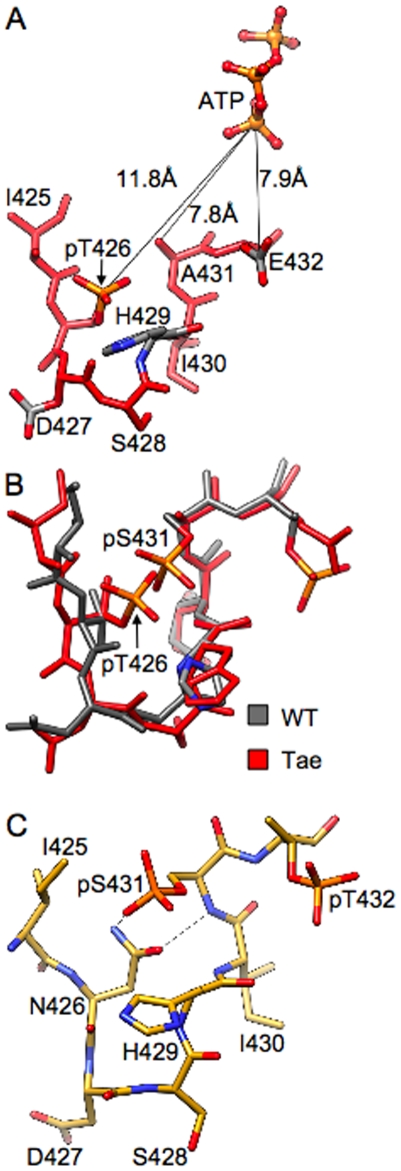
Phosphorylation of T426 in selected subunits of the KaiC^Tae^ double mutant. (**A**) Conformation of the P-site loop region encompassing residues I425 to E432 in the A subunit. Distances between the γ-phosphorus of ATP and the Cδ (E432), Cβ (A431) and phosphorus (pT426) positions (marked by thin solid lines) are shown in Å. (**B**) The hypothetical, simultaneous phosphorylation at S431 and T426 would lead to a clash. The superimposition shows the P-site loops in subunits A of the wt-KaiC and Tae mutant structures. (**C**) Hydrogen bonding interaction (dashed lines) between N426 and pT431 in the crystal structure of the KaiC^nST^ mutant.

The crystal structures of the KaiC^TaT^ and KaiC^Tae^ mutants are noteworthy in two respects. For one, the spacing between 431 residues (Oγ) and the γ-phosphate is tighter than that between 432 residues and Pγ (**Table 2**). In addition, inspection of the electron density reveals that four of six T426 residues carry a phosphate group in the crystal of KaiC^Tae^ (absent in subunits C and D; **Fig. 5A**). In the KaiC^TaT^ structure, T426 from subunit A also carries a phosphate group. These structures demonstrate for the first time that T426 can become phosphorylated, although it is farther removed from ATP than either the 432 or 431 residues (**Table 2**; however, the average distance of 11.9 Å between pT426 and the γ-phosphate is not dramatically different from the 9.4 Å distance for pS431 residues in the structure of wt-KaiC). Because the double mutant KaiC^Tae^ can be considered a model system for the second phosphorylation step, we are assuming that the inability of transferring the phosphate to A431 triggers phosphorylation of T426. Although the side chain of A431 (methyl) is shorter than the hydroxymethylene moiety in wt-KaiC the structural data leave no doubt that S431 and T426 cannot both be phosphorylated. The loop harboring the two residues is too tight at that location and attaching a phosphate group to both S431 and T426 would lead to a clash (**Fig. 5B**). The earlier finding that the aST mutant is arhythmic [21] and the *in vivo* data presented in the accompanying paper [28] that reveal that the nST mutant, although capable of forming a hydrogen bond to pS431 (**Fig. 5C**), does not restore wt function dovetail with our structural data. Instead of S431 and T426 residues being phosphorylated simultaneously, a much more likely scenario may entail the phosphate shuttling between the two. Thus, the phosphate is mainly bound to S431 but can occasionally jump to T426, thus prolonging the lifetime of phosphorylation there relative to T432. This idea is supported by the significantly longer half-life of the TpS band during the KaiB-assisted dephosphorylation and subunit-exchange phase in SDS-PAGE assays of the *in vitro* oscillator [24). Moreover, the observation that KaiB is unable to antagonize KaiA in the case of the aST and nST mutants [28] supports a role of residue 426 in the dephosphorylation step.

### Structure-Based Insights into the Kinase and ATPase Mechanisms

**Figure 6:**
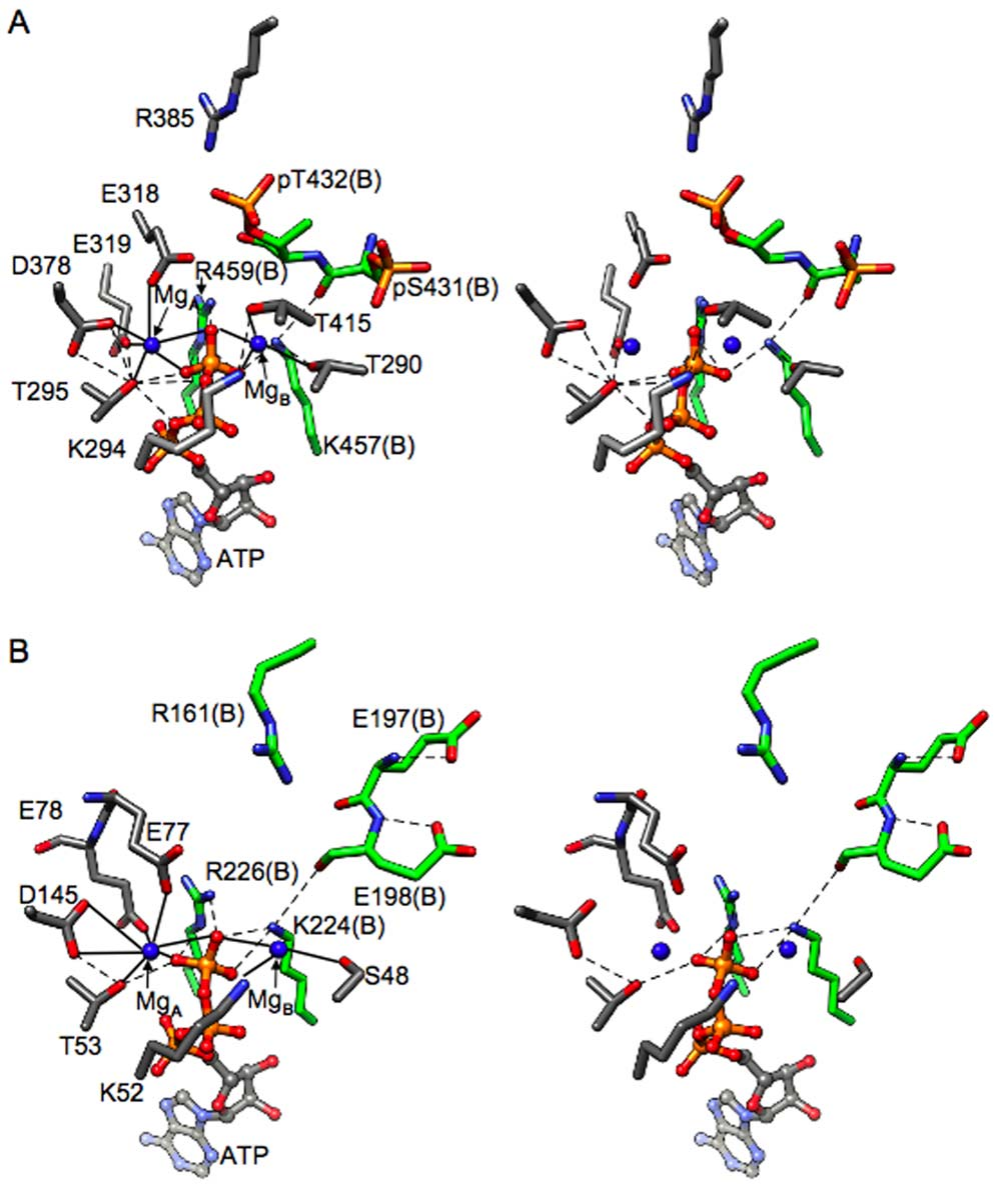
The kinase and ATPase mechanisms. Stereo diagrams depicting the active sites in the (**A**) KaiCII and (**B**) KaiCI halves of KaiC^nST^. Carbon atoms of residues from adjacent subunits are colored in gray and green (subunits A and B, respectively), Mg^2+^ ions are depicted as blue spheres with coordination geometries indicated by bold lines and hydrogen bonds are dashed lines.

Based on the structures of wt-KaiC and six mutants and in conjunction with data from site-specific mutations (**Fig. 3**), the divergent functions of the CI and CII halves in the control of the KaiABC oscillator and the roles of individual residues underlying the mechanisms of the kinase (CII half) and ATPase (CI and CII halves) activities can be revisited. Comparisons between the distinct configurations and identities of amino acids in the vicinity of ATP phosphate groups in the CII and CI halves are depicted in **Fig. 6**. Pairs of glutamates in the CI and CII halves (E77/78 and E318/319, respectively) take on key roles in the conversion of ATP to ADP (see **Fig. S1**, supporting information, for an alignment of the CI and CII sequences). In CII, E318 is the catalytic glutamate [5] that activates the T432 and S431 hydroxyl groups for nucleophilic attack at the γ-phosphate group. Both the E318A and E318D mutants are arhythmic and the former lacks the ability for phosphoryl transfer judging from SDS-PAGE gels (**Fig. 3**). Three basic residues, K294(n), K457(n+1) and R459(n+1) stabilize the negative charges on the phosphates and lock the ATP molecule in place. E319 is involved in the coordination of one of the two Mg^2+^ ions found at the active site (Mg^2+^ ion A, **Fig. 6A**). Mg^2+^ ion A is bound between the γ-phosphate and the Thr/Ser residues that become phosphorylated and shepherds the nucleophile toward the former. Mg^2+^ ion B is bound between the γ- and β-phosphates and acts as a Lewis acid to stabilize the pentacovalent transition state and facilitate departure of the oxyanion. Therefore, the kinase relies on the ubiquitous two-metal-ion phosphoryl-transfer mechanism ([29] and cited refs.). The coordination of metal cations involves a mixture of the inner- and outer-sphere modes, but the resolutions of the diffraction data (**Table 1**) are not sufficient to allow a reliable assignment of water molecules coordinated to Mg^2+^ ions. In CI, E77 takes on the role of deprotonating a water molecule and thus activating it for ATP hydrolysis. As in the CII half, two lysines [K52 and K224(n+1)] and an arginine [R226(n+1)] stabilize the orientation of the ATP phosphates (**Fig. 6B**). Residue E78 is coordinated to Mg^2+^ ion B, but a third glutamate (E183 not shown in **Fig. 6B**) is also situated in close vicinity from the γ-phosphate [Cδ…γP≈6.5 Å (E78)/7.5 Å (E183)/8.5 Å (E77)]. Based on the structure alone and in the absence of site-specific mutational data, it is unclear what the role of E183 might be.

In the CII half, R385, that forms an inter-subunit salt bridge to T432 once the latter becomes phosphorylated (see above), also lies in close vicinity of the general acid/base E318 (**Fig. 6A**). Interestingly, the R385A mutant is hyper-phosphorylated, possibly indicating that the lack of the R385…E318 interaction facilitates activation of T432 and S431 by E318 and thus increases phosphorylation (**Fig. 3**). Alternatively, since R385 interacts with pT432 across the subunit interface, the R385A mutation could somehow affect dephosphorylation. However, this latter hypothesis is not supported by an *in vitro* dephosphorylation assay, as the data do not indicate diminished phosphatase activity in the KaiC^R385A^ mutant relative to wt-KaiC (**Fig. S2**, supporting information).

Although both the CI and CII halves exhibit ATPase activity, phosphorylation across subunits suppresses the ATPase activity [30]. This interpretation is consistent with the increased ATPase activity of the Taa mutant relative to wt-KaiC; once dephosphorylated the ATPase activity resumes. Unlike the CII half that exhibits kinase, phosphatase and ATPase activities, the CI ring cannot act as an auto-kinase or auto-phosphatase. A comparison between the loop region harboring the T432, S431 and T426 sites in CII and the equivalent region in CI provides a rationalization for these differences. The residues in CI corresponding to the T432, S431, and T426 sites in CII are E198, E197 and A192, respectively (**Fig. S1**). Thus, the loop in CI mimics a hyper-phosphorylated state, depriving a potential kinase activity of target sites. Further it is remarkable that CI residue 192 that corresponds to T426 in CII is an alanine. We believe that the presence of Ala rather than Thr or Ser at this site is to rule out a phosphoryl transfer in the CI half, thus limiting the roles of the N-terminal KaiC ring to catalyze ATP hydrolysis and serve as a structural platform.

### Residues Flanking the T432, S431 and T426 P-Sites Form Part of a Hydrophobic Core

**Figure 7:**
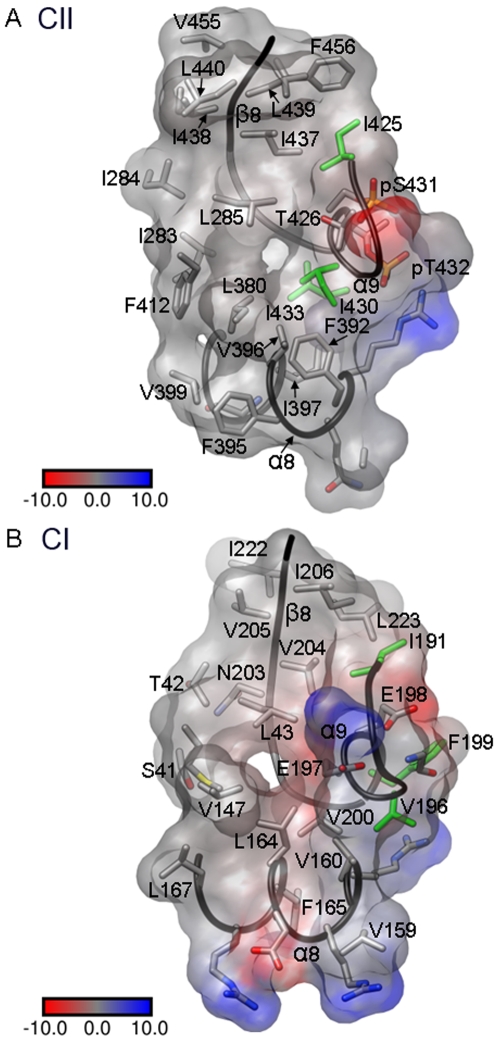
A hydrophobic pocket anchors isoleucine residues adjacent to P-sites in CII. (**A**) Residues flanking the T432, S431 and T426 phosphorylation sites in CII (I433, I430 and I425, respectively; side chains highlighted in green) point into a hydrophobic patch. (**B**) Similarly, residues corresponding to CII P-sites in CI (E198, E197 and A192) are flanked by hydrophobic residues (F199, V196 and I191, respectively; side chains highlighted in green). However, judging from the calculated electrostatic surface potential [39], the pocket in CI appears to be slightly more polar compared with CII.

In the CII half, the residues that flank the phosphorylation sites (425, 430 and 433) are all isoleucines. The Ile side chains point away from ATP (**Fig. 7A**) and participate in an extended network of hydrophobic interactions that involve no fewer than twelve Ile, Val and Phe residues (**Fig. S1**). To assess the effects of a mutation of one of the above Ile residues on phosphorylation and clock rhythm we generated the I430A mutant. This mutation renders the clock arrhythmic and the SDS-PAGE analysis indicates that KaiC^I430A^ is hyper-phosphorylated (**Fig. 3**). The thermodynamic stability of the I430A mutant is reduced relative to wt-KaiC; CD melting experiments indicate that the T_m_ of the mutant is lowered by about 3°C relative to wt-KaiC. In the CI half a similar sequence pattern exists in that the residue adjacent to E198 is Phe (F199), the one next to E197 is Val (V196) and the neighbor of A192 is Ile (I191) (**Figs. S1**, **7B**). However, the environment of the loop region in CI is somewhat less hydrophobic compared with CII as manifested by a more negative electrostatic surface potential (**Fig. 7**). Based on the available observations the mutation of I430 to Ala most likely triggers a change in the mobility of the P-site loop that severely distorts the balance between the hypo- and hyper-phosphorylated forms.

## Discussion

Among the structures of the six KaiC mutant proteins reported here, those of the TaT and Tae mutants are the most intriguing because they imply that T426 can be a third phosphorylation site. The observation of a phosphate at T426 in the crystals but not under other conditions, i.e. SDS-PAGE assays using heated KaiC^Tae^ samples [28] or mass spectrometric analyses of wt-KaiC [22], [24] may indicate that phosphorylation at this site is labile. However, we believe that the conditions for growing KaiC crystals promote increased phosphorylation levels relative to protein isolated from *in vitro* cycling reactions or incubated with ATP at elevated temperatures. For example, as compared with the two latter procedures, the crystallization conditions replace ATP with ATPγS and involve a lower pH (<5). The kinase activity of KaiC is enhanced under acidic conditions [6], resulting in transfer of ATPγS thiophosphate groups to T432, S431 or T426. In this context it is noteworthy that crystals of KaiC grown at neutral pH and/or in the presence of ATP are inferior to those obtained at low pH with ATPγS. Once thiophosphate groups have been transferred to CII Thr and Ser residues they will likely stay bound as thio-phosphorylated KaiC may be resistant to auto-dephosphorylation, similar to what has been reported for CaM kinase II [31]. This will keep the KaiC hexamer in a stable state of elevated phosphorylation that is presumably conducive to crystallization instead of producing a mixture of KaiCs in various states of phosphorylation when ATP is present. Therefore, phosphorylation at T426 seen in selected subunits of the Tae and TaT mutants is most likely not a crystallographic artifact. Rather the crystal structures have preserved a third P-site in KaiCII that appears to have evaded identification by other approaches.

**Figure 8:**
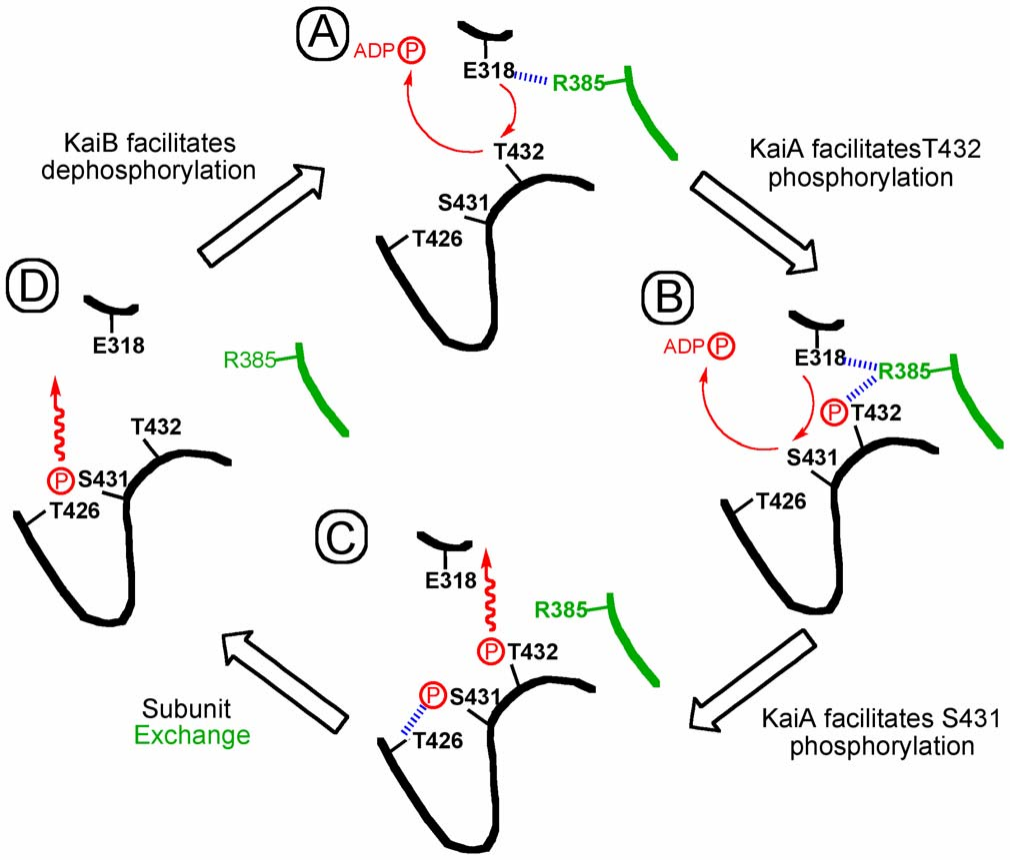
Sequence of the phosphorylation and dephosphorylation events over the 24-hour cycle of the KaiABC clock and the role of T426. The P-site loop along with the catalytic residue E318 from one KaiC subunit and a portion of the α7 helix (R385) from the adjacent subunit are colored in black and green, respectively. Hydrogen bonds are blue, activation of T432 and S431 by E318 as well as phosphoryl transfers are indicated by red arrows, and a wiggly red arrow implies phosphatase activity.

The T426 phosphorylation site is not a substitute for S431 as demonstrated by the arhythmic behavior of the TaT and aST mutants [21]. Clearly, both sites have to be phosphorylatable as the nST mutant that exhibits hydrogen bond formation between the Asn side chain and the 431 phosphoserine (**Fig. 5C**) is also arhythmic. However, the combined experimental evidence also supports the conclusion that S431 is directly phosphorylated (not T426) following phosphorylation at T432 (see **Fig. 8** for a cartoon of the phosphorylation and dephosphorylatrion events over a 24-hour cycle). For example, in the structure of wt-KaiC, all subunits display phosphorylation at T432 and four subunits feature a phosphoserine at position 431 [4], [18], [21]. By comparison, only one subunit is phosphorylated at T426 in the TaT mutant (this work). The lower level of phosphorylation there correlates with the longer distance between the Cα of residue 426 and ATP Pγ compared with the corresponding distance for Cα of residue 431 (**Table 2**). T426 likely serves an auxiliary role and the observations described in the accompanying paper [28] point to an involvement in the dephosphorylation step of the clock cycle (**Fig. 8**). Thus, the nST mutant slows considerably the rate of dephosphorylation and both the aST and nST mutants prevent KaiB from antagonizing KaiA's action [28].

We have already suggested a “ratcheting” mechanism for the phosphorylation step that can explain the unidirectional nature of the phosphorylation sequence on the basis of increased molecular interactions at the subunit interface [4]. The structural data indicate proximity to the ATP γ-phosphate as the likely reason for T432 being phosphorylated before S431 (**Table 2**). What then determines the order of the dephosphorylation steps? The KaiC crystal structures reveal different environments of the phosphates at T432 and S431 with the former involved in inter-subunit contacts and the latter contained in a single subunit (**Figs. 1**, **8**). KaiB binding and subunit shuffling [25]–[27] likely destabilize the phosphothreonine at 432 to a larger extent than the phosphoserine at 431, particularly if we consider that 431 could share its phosphate with T426 (**Figs. 5A,B** and **8**). Thus, the different environments of 432 and 431, with pT432 being more exposed and a threonine residue in the immediate vicinity of pS431 can potentially explain the order of dephosphorylation. Not only does T426 stabilize phosphorylation at S431 via hydrogen bonding (**Fig. 1B**), but the phosphate may be shuttling between the two (**Fig. 5B**), with T426 also involved in dephosphorylation of pS431 [28]. The latter role is in line with the interactions between pS431 and the side chains of 426 residues in the crystal structures (**Figs. 1**, **5**). Moreover, the hampered ability of KaiB to antagonize KaiA with the KaiC^aST^ and KaiC^nST^ mutants could be due to their altered electrostatic surface potentials relative to wt-KaiC as a result of the lack of a phosphorylatable residue at position 426.

The crystal structures described here manifest only minor conformational changes in the P-site loop region as a result of single or double mutation (**Fig. 4**). The distances between P-sites and the γ-phosphate of ATP (**Table 2**) argue against large conformational changes at the subunit interface for phosphoryl transfer. However, the starkly different phosphorylation profiles and period lengths of KaiCs with mutations in the P-site loop region and adjacent residues (**Fig. 3**) demonstrate that not every residue in the loop plays an important role. Whereas the T426A and I430A mutants are arhythmic, the H429A mutant exhibits a slightly longer period but a phosphorylation profile that is very similar to that of wt-KaiC (**Fig. 3**). This is despite the fact that H429 hydrogen bonds to pS431 (**Fig. 1B**). Similarly, mutations of D417 and D427 (not shown) to alanine are of little consequence although they lie in the immediate vicinity of S431 (**Fig. 1B**) and T426, respectively. Therefore, these comparisons highlight the functional importance of the T426 residue [28]. The evidence presented here for a role of T426 in the dephosphorylation step and R385 (located adjacent to the kinase active site) critically affecting the balance between the hyper- and hypo-phosphorylated states also argues against a large spatial separation of the auto-kinase and auto-phosphatase activities in KaiC.

## Methods

### Protein Expression and Purification

*S. elongatus* KaiC with a C-terminal (His)_6_-tag was produced in *E. coli* (BL21, DE3 cell line) as previously described [14], [18]. Site-directed mutagenesis was performed with the QuikChange® XL Site-Directed Mutagenesis system (Stratagene, La Jolla, CA) and all mutant proteins were expressed following the protocol used with wt-KaiC. KaiC proteins were purified by metal affinity chromatography (TALON IMAC resin, BD Biosciences Clontech) and then by gel filtration chromatography (Sephacryl S-300 HR resin, Amersham Biosciences). The solutions of purified proteins were concentrated (10∼20 mg/mL) and ATP in the buffer was replaced with ATPγS by ultrafiltration for crystallization.

### Crystallization and Diffraction Data Collection

Crystals of mutant proteins were grown using conditions previously established for wt-KaiC [18]. Crystals were mounted in nylon loops, cryo-protected in 25% glycerol containing reservoir solution and frozen in liquid nitrogen. Diffraction data were collected on 21-ID beam lines of the Life Sciences Collaborative Access Team (LS-CAT) at the Advanced Photon Source, Argonne National Laboratory (Argonne, IL) using either MarMosaic 225 or MarMosaic 300 CCD detectors. All diffraction data were integrated and scaled with either the HKL2000 [32] or XDS [33] programs. Mutant structures were determined with the Molecular Replacement technique using the program CNS [34] and the wt-KaiC structure with PDB ID 3DVL [19] as the search model. Initial refinement was carried out with the program CNS and mutations were gradually built into the electron density, followed by further positional and isotropic B-factor refinement. Manual rebuilding was performed with the programs TURBO [35] and COOT [36]. Water molecules were added gradually and positional and isotropic B-factor refinement cycles were continued with the program CNS. A summary of crystallographic parameters is provided in **Table 1**. All illustrations were generated with the program CHIMERA [37].

### Dephosphorylation Assays

The R385A substitution was introduced by site-directed mutagenesis into the plasmid pGEX-6P-1 carrying the wild-type *kaiC* ORF [9]. The wild-type and R385A KaiC proteins were expressed in *E. coli* and purified as described [22] with minor modifications. Purified KaiC proteins (0.2 µg/µL) were incubated at 30°C in 20 mM Tris-HCl, 150 mM NaCl, 5 mM MgCl_2_, 1 mM ATP, 0.5 mM EDTA, 8.4% (v/v) glycerol, pH 8.0 in the absence of KaiA or KaiB. Phosphorylation states of KaiC proteins were examined by SDS-PAGE as described in [28].

### *In Vitro* Luminescence Rhythm and KaiC Phosphorylation

To introduce nucleotide substitutions responsible for E318A, E318D, R385A, D417A, H429A, and I430A into the *kaiC* gene, the PCR-based *in vitro* mutagenesis was performed with pC*kaiABC* [5] as a template. The pC*kaiABC* derivatives were introduced into a *kaiABC*-deleted (Δ*kaiABC*) strain carrying a *kaiBC*p::*luxAB* reporter gene set [22]. The *kaiBC* promoter activity in each mutant strain was monitored as bioluminescence [38]. To assess the effects of mutations on KaiC phosphorylation *in vivo*, mutant cells were grown in liquid BG-11 medium under cycles of 12-h light and 12-h darkness, harvested at ZT4, and subjected to immunoblot analysis with anti-KaiC antibodies.

### Deposition of Atom Coordinates

All structure factors and final coordinates have been deposited in the Protein Data Bank (www.rcsb.org); the PDB ID codes are: 3jzm (TSa KaiC), 3k0a (TaT KaiC), 3k09 (TdT KaiC), 3k0e (nST KaiC), 3k0f (aSa KaiC), and 3k0c (Tae KaiC).

## Supporting Information

Figure S1 Sequence alignment of the KaiC CI (top) and CII (bottom) halves. Secondary structural elements are indicated by cylinders (α-helices) and arrows (β-strands) above the sequences. P-site loop residues including T426, S431 and T432 (CII) and the corresponding residues A192, E197 and E198 (CI) are boxed. Asterisks designate hydrophobic residues from the α 6, α 8, β 7, β 8 and β 9 regions that anchor the I425 (I191, CI), I430 (V196, CI) and I433 (F199, CI) residues of the P-site loop (CII, Fig. 7).
(0.75 MB TIF)

Figure S2 The hyper-phosphorylated phenotype of the R385A mutant in vivo is likely due to increased auto-kinase activity rather than diminished auto-phosphatase activity. Hyper-phosphorylated wt-KaiC and R385A KaiC mutant proteins were incubated at 30C in the absence of KaiA or KaiB for up to 48 hours and the ratio of the amount of each phospho-form of KaiC to the total amount was determined at the indicated times by densitometric analysis of CBB-stained PAGE gels. T and S refer to the T432 and S431 residues, respectively. The analysis indicates similar distributions of the various KaiC forms over time, the only apparent differences being higher initial levels of the phosphorylated forms with the R385A mutant and a slightly increased level of the pS/T form for the mutant relative to wt-KaiC after 24 hours. These data support the conclusion that the R385A mutant does not hamper dephosphorylation but that the hyper-phosphorylated phenotype (please see Fig. 3) is probably due to increased kinase activity.
(7.25 MB TIF)
