# Supplementary figures and images for: Structures of KaiC Circadian Clock Mutant Proteins: A New Phosphorylation Site at T426 and Mechanisms of Kinase, ATPase and Phosphatase
Source: PLoS One. 2009 Nov 26;4(11):e7529. doi: 10.1371/journal.pone.0007529 (PMC2777353; doi:10.1371/journal.pone.0007529)

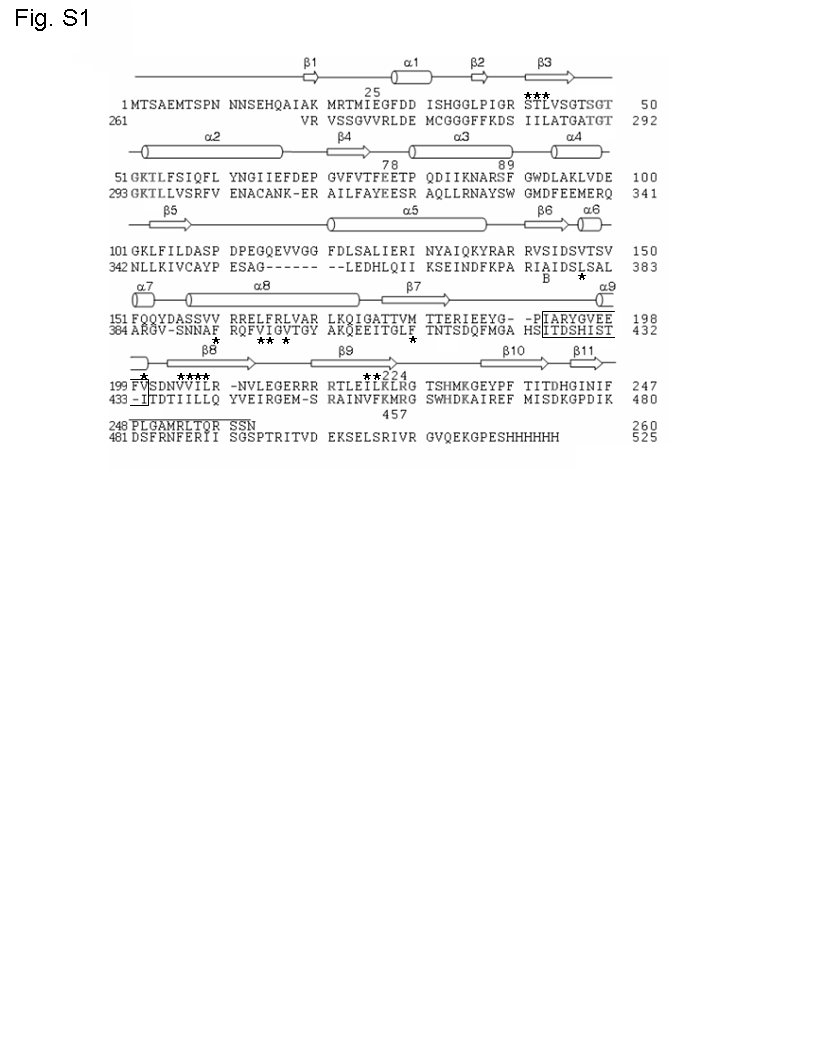

Supplement: Figure S1 — Sequence alignment of the KaiC CI (top) and CII (bottom) halves. Secondary structural elements are indicated by cylinders (α-helices) and arrows (β-strands) above the sequences. P-site loop residues including T426, S431 and T432 (CII) and the corresponding residues A192, E197 and E198 (CI) are boxed. Asterisks designate hydrophobic residues from the α 6, α 8, β 7, β 8 and β 9 regions that anchor the I425 (I191, CI), I430 (V196, CI) and I433 (F199, CI) residues of the P-site loop (CII, Fig. 7). (0.75 MB TIF) [file pone.0007529.s001.tif]

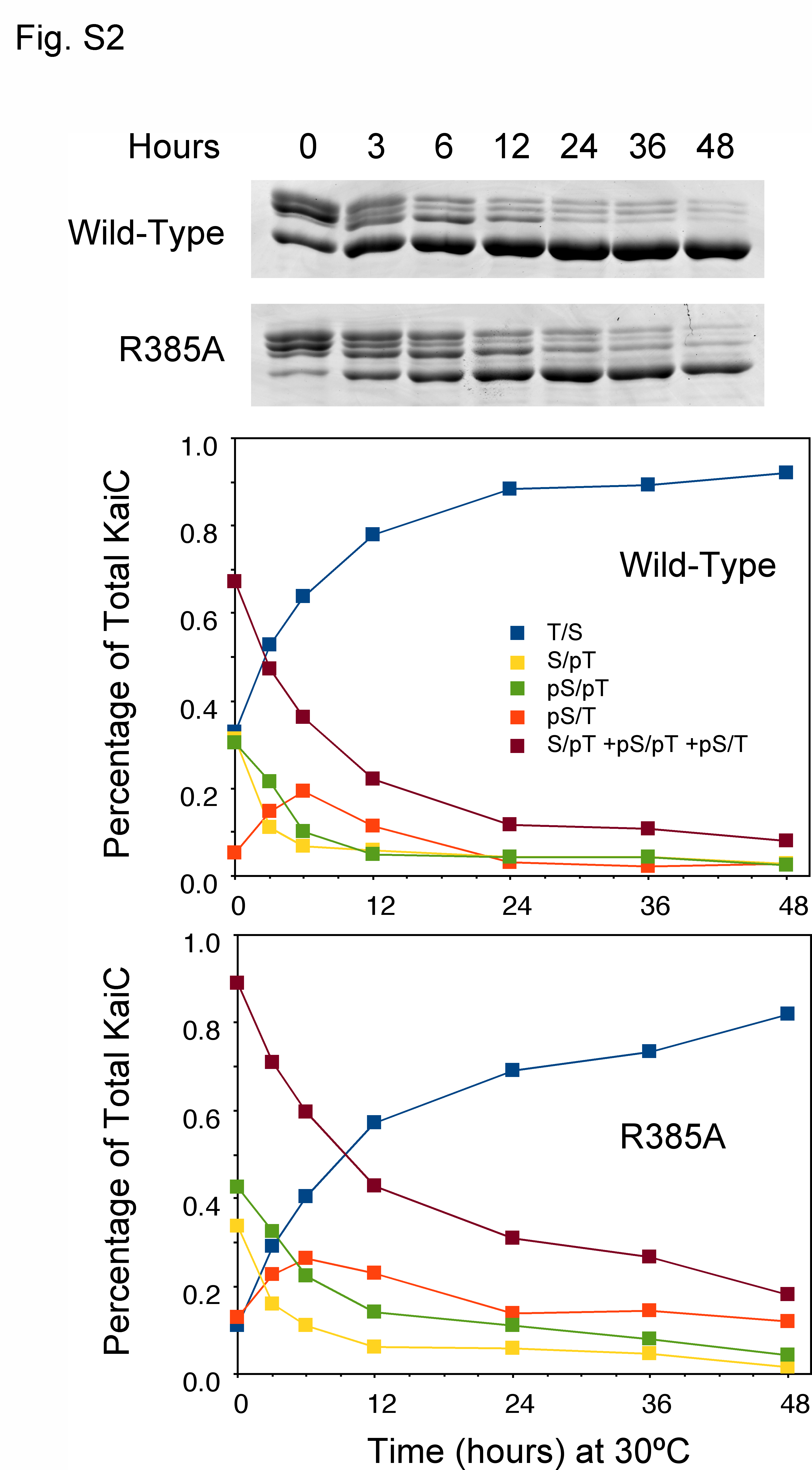

Supplement: Figure S2 — The hyper-phosphorylated phenotype of the R385A mutant in vivo is likely due to increased auto-kinase activity rather than diminished auto-phosphatase activity. Hyper-phosphorylated wt-KaiC and R385A KaiC mutant proteins were incubated at 30C in the absence of KaiA or KaiB for up to 48 hours and the ratio of the amount of each phospho-form of KaiC to the total amount was determined at the indicated times by densitometric analysis of CBB-stained PAGE gels. T and S refer to the T432 and S431 residues, respectively. The analysis indicates similar distributions of the various KaiC forms over time, the only apparent differences being higher initial levels of the phosphorylated forms with the R385A mutant and a slightly increased level of the pS/T form for the mutant relative to wt-KaiC after 24 hours. These data support the conclusion that the R385A mutant does not hamper dephosphorylation but that the hyper-phosphorylated phenotype (please see Fig. 3) is probably due to increased kinase activity. (7.25 MB TIF) [file pone.0007529.s002.tif]
